# Prediction of post-extubation failure by portable ICU ultrasound

**DOI:** 10.1186/cc10737

**Published:** 2012-03-20

**Authors:** Y Sutherasan, P Theerawit, T Hongpanat, C Kiatboonsri, S Kiatboonsri

**Affiliations:** 1 Ramathibodi Hospital, Mahidol University, Bangkok, Thailand

## Introduction

Stridor and vocal cord oedema are common in ICU patients. Currently, the cuff leak volume test is a standard technique to assess these complications [1,2]; however, wide variations in terms of its sensitivity and specificity have been demonstrated in many studies. Recently, ultrasound is a promising noninvasive method widely used in ICU patients and allows visualization of the vocal cords and larynx [3]. Thus, we would like to determine the diagnostic accuracy of portable ultrasound for detection of these post-extubation complications.

## Methods

We conducted a prospective, observational study from December 2010 to September 2011 using portable critical care ultrasound to examine air-column width differences of vocal cords before and after deflation of a endotracheal cuff balloon. All patients also underwent cuff leak volume tests and vocal cord examination by direct video laryngoscopy.

## Results

We enrolled 101 patients with planned extubation. The overall prevalence of post-extubation stridor and/or vocal cord oedema was 17%. Age, gender, duration of intubation and BMI were not different between patients with and without post-extubation complications. The average sizes of endotracheal tubes were similar in both groups (No. 7.5). The mean difference of increasing of air-column width in patients without complications was considerably higher than those with complications (1.9 mm vs. 1.1 mm; *P *< 0.001). The sensitivity and specificity at air-column width differences ≥1.6 mm were 0.706 and 0.702 respectively. The positive predictive value and negative predictive value were 0.324 and 0.922. The area under the ROC curve of tracheal ultrasound was 0.823 (95% CI: 0.698 to 0.947) and that of the cuff leak volume test was 0.840 (95% CI: 0.715 to 0.964).

## Conclusion

Portable ICU ultrasound visualising air-column width differences between pre and post deflation cuff balloon is a promising objective tool which aids in prediction of successful extubation.
